# A Quantitative Comparison of Overlapping and Non-Overlapping Sliding Windows for Human Activity Recognition Using Inertial Sensors

**DOI:** 10.3390/s19225026

**Published:** 2019-11-18

**Authors:** Akbar Dehghani, Omid Sarbishei, Tristan Glatard, Emad Shihab

**Affiliations:** 1 Department of Computer Science and Software Engineering, Concordia University, Montreal, QC H3G 1M8, Canada; a_ehg@encs.concordia.ca (A.D.); tristan.glatard@concordia.ca (T.G.); 2 Research and Development Department, Motsai Research, Saint Bruno, QC J3V 6B7, Canada; o.sarbishei@motsai.com

**Keywords:** activity recognition, inertial sensors, supervised classification

## Abstract

The sliding window technique is widely used to segment inertial sensor signals, i.e., accelerometers and gyroscopes, for activity recognition. In this technique, the sensor signals are partitioned into fix sized time windows which can be of two types: (1) non-overlapping windows, in which time windows do not intersect, and (2) overlapping windows, in which they do. There is a generalized idea about the positive impact of using overlapping sliding windows on the performance of recognition systems in Human Activity Recognition. In this paper, we analyze the impact of overlapping sliding windows on the performance of Human Activity Recognition systems with different evaluation techniques, namely, subject-dependent cross validation and subject-independent cross validation. Our results show that the performance improvements regarding overlapping windowing reported in the literature seem to be associated with the underlying limitations of subject-dependent cross validation. Furthermore, we do not observe any performance gain from the use of such technique in conjunction with subject-independent cross validation. We conclude that when using subject-independent cross validation, non-overlapping sliding windows reach the same performance as sliding windows. This result has significant implications on the resource usage for training the human activity recognition systems.

## 1. Introduction

Wearable sensors and mobile devices are transforming society at a rapid pace, creating a wide range of opportunities for knowledge extraction from new data sources. Human Activity Recognition (HAR), in particular, is an active research area due to its potential applications in security, virtual reality, sports training, and health care. For instance, HAR has been used to detect anomalous behaviors such as falls [1] and track movement-related conditions in seniors [2].

Most HAR systems use an Activity Recognition Process (ARP) to detect activities. These systems usually consist of one or more inertial sensors attached to different parts of a person’s body that provide diverse streams of sensor data. Such data streams, subsequently, are segmented into several time windows with specific lengths and from which feature vectors are extracted and fed to a classifier. Segmentation in time windows is a critical process in ARP, often implemented with sliding windows [3,4] that can be either overlapping or non-overlapping [5]. The literature contains a plethora of examples where both overlapping (e.g., [6,7,8,9]) and non-overlapping time windows are used (e.g., [4,10,11,12]).

### 1.1. Overlapping Sliding Windows

The work by Ling Bao and Stephen S. Intille [6] uses overlapping sliding windows to segment signals coming from five biaxial accelerometers placed on 20 subjects (13 males and seven females) under laboratory and semi-naturalistic conditions while performing 20 daily activities. Subsequently, each window is transformed into a set of features namely mean, energy, frequency-domain entropy, and correlation of acceleration data. The authors use k-nearest neighbor (KNN), decision tree (DT), and naive Bayes (NB) classifiers, and subject-independent cross-validation (CV) and subject-dependent CV for system evaluation. They reach the overall accuracy of 84% with DT under the subject-independent CV process.

Another example is the work by Tapia et al. [7] where the authors develop a real-time recognition system to recognize physical activities and in some cases, their intensities. They segment the signal data collected from 21 subjects wearing triaxial wireless accelerometers and a wireless heart rate monitor while performing 30 physical gymnasium activities using overlapping sliding windows. Subsequently, from each window, they extract time domain and frequency domain features and using a DT classifier they recognize activities with an accuracy of 94.6% using subject-dependent CV and 56.3% using subject-independent CV.

Lara et al. [8] combine acceleration data with vital signs to improve the performance of HAR systems. They apply ARP on a dataset which was collected from eight subjects (seven males and one female) while wearing a BioHarness™ BT chest sensor strap [13]. Using data from a triaxial accelerometer and vital signs, they detect five activities including running, walking, sitting, ascending, or descending. Sensor signals are partitioned into overlapping time windows with three different sizes: 5 s, 12 s, and 20 s sliding at 50% of their sizes and 90 features were extracted from each time window. NB, DT, Bayesian Network, Multilayer Perceptron, Additive Logistic Regression and classifier ensembles are used to recognize activities. They achieve up to 95.7% overall accuracy, which was evaluated through subject-dependent CV. Their results also indicate that vital signs are useful to discriminate among certain activities like running and sitting compared to the cases that utilize acceleration data only.

Recofit [9] is a well-known reference on HAR, which applies ARP on a dataset of accelerometer and gyroscope data collected from 114 participants over 146 sessions. The authors address three major challenges namely (1) segmenting exercise from intermittent non-exercise periods, (2) recognizing which exercise is being performed, and (3) counting repetitions. Data points are windowed into 5 s overlapping windows sliding at 200 ms and subsequently, each window is transformed into 224 features. Linear support vector machines (SVM) are used in the classification stage and evaluated by subject-independent CV. Spectacular performance is achieved, with precision and recall greater than 95% to identify exercise periods, recognition of up to 99% for circuits of 4 exercises, and counting accurate to ±1 repetition, 93% of the time.

### 1.2. Non-Overlapping Sliding Windows

Regarding non-overlapping windowing, Minnen et al. [10] describes an activity recognition component to recognize soldier activities as a part of the Soldier Assist System (SAS). They apply ARP on the signal of a six three-axis bluetooth accelerometers positioned on the right thigh sidearm holster to recognize 14 soldier activities. The sensor signals are partitioned by 3 s non-overlapping sliding windows and then each window is transformed into 378 features. A boosting ensemble classifier is used to select the most important features and also recognize the activities. Their recognition system achieves 78.7% for continuous event recognition (considering null activity) and 70.3% frame level accuracy. These values increase to 90.3% and 90.3%, respectively when considering only the modeled activities. In their study, they use subject-independent CV to evaluate their system.

Another example is the work by REDDY et al. [11], where the authors create a transportation mode recognition system using a mobile phone to identify whether an individual is stationary, walking, running, biking, or in motorized transport. The dataset used in their study contains accelerometer measurements, GPS, WiFi, and GSM signals of sixteen individuals (eight male and eight female) while six phones attached to their bodies simultaneously and were in one of the five transportation modes for fifteen minutes. The signals are windowed into 1 s non-overlapping sliding windows and each window is transformed into a set of features such as magnitude of the force vector, mean, variance, energy, etc. They use several classifiers namely DT, NB, KNN, SVM, Hidden Markov Model and a two stage classifier involving DT combined with discrete Hidden Markov Model. They achieve an accuracy level of 93.6% with the two stage classifier, which was evaluated by subject-dependent CV.

Cheng et al. [12] implement an on-body capacitive sensing approach to recognize activities such as chewing, swallowing, speaking, sighing (taking a deep breath), as well as different head motions and positions. They use a dataset that contains the 4.3 h-electrode collar data which was collected from three subjects (one female, two males; aged between 25 and 45 years) while performing a set of head movements, swallow water from a cup, chew and swallow bread pieces and speak. Each signal is partitioned into 1.5 s non-overlapping sliding windows and each window then is transformed into time domain features such as signal mean, variance, maximum, etc. They use a linear discriminant classifier to identify various activities and evaluate the system through subject-dependent CV and report the accuracy rate for the combination of activities.

Another example is the work by Banos et al. [4], where the authors present an extensive study to distinguish the windowing procedure and its impacts on the recognition system. They apply ARP on the accelerometer data of a benchmark dataset collected from 17 subjects of different profiles performing 33 fitness activities in an out-of-lab environment. Sensor signals are windowed into non-overlapping windows with a substantial set of window sizes ranging from 0.25 s to 7 s in steps of 0.25 s. Each window is then transformed into three different feature sets (FS) namely FS1 (mean only), FS2 (mean and standard deviation) and FS3 (mean, standard deviation, maximum, minimum and mean crossing rate). They use DT, KNN (K = 3), NB and Nearest Centroid Classifier (NCC) as the classifiers and subject-dependent CV for system evaluation. From this study, they prove that the interval 1–2 s is the best trade-off between recognition speed and performance. Besides, they provide a set of guidelines for system definition and configuration based on the particular application requirements and target activities.

### 1.3. Our Contributions

As summarized previously, several works have shown that using overlapping sliding windows instead of non-overlapping ones improves the accuracy of the recognition systems. However, the amount of such improvement and its sources in HAR remain unclear. This study addresses this question: based on a detailed, quantitative analysis of multiple datasets, we explain why and by how much overlapping windows affect the performance of ARP. We report and discuss the general and per activity impacts of such two methods considering two validation techniques namely subject-dependent CV and subject-independent CV.

The main contributions of our work are:

- An in-depth investigation of how HAR system performance is impacted by overlapping and non-overlapping sliding windows.
- A set of publicly available scripts [14] to help the research community further shed light on the important topic of choosing the types of sliding windows in HAR.

The rest of the paper is structured as follows. Section 2 describes background in ARP, system validation, and sliding windows. In Section 3 we explain our ARP setting, and in Section 4, we present our results. Finally, we discuss the results in Section 5, and we summarize our conclusions in Section 6.

## 2. Background

In this section, we provide an overview of a typical activity recognition process (ARP), we explain and we compare different sliding windows techniques, and we describe common evaluation methods in HAR.

### 2.1. Activity Recognition Process

**Figure 1:**
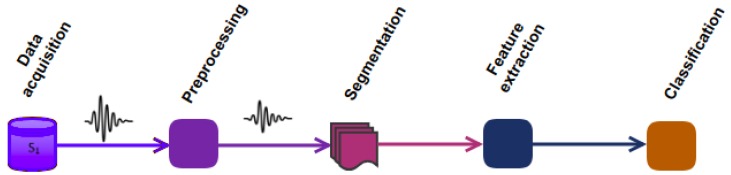
Human activity recognition process.

ARP is composed of a sequence of signal processing, pattern recognition, and machine learning techniques [15]. It mainly consists of 5 steps, shown in Figure 1 and explained hereafter.

**Figure 2:**
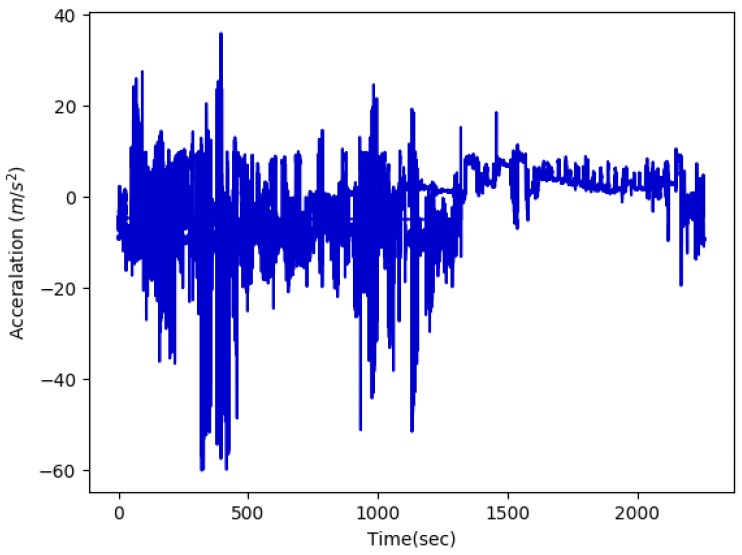
Example acceleration data extracted from [16].

**Data acquisition.** Several sensors are attached to different body parts. They mostly acquire 3D acceleration, gyroscopic and magnetic field measurements, as shown in Figure 2. Sensors discretize signals at a given frequency, typically 50 Hz for daily activities or 200 Hz for fast sports, and transmit the resulting data points to the receiver.

**Pre-processing.** Data points coming from sensors may include artifacts of various origins such as electronic fluctuations, sensor malfunctions, and physical activities [17]. To eliminate such artifacts, filtering techniques are commonly applied, such as the Butterworth low-pass filter, which attenuates the higher frequency components of the signal beyond a configurable cut-off frequency [9,18,19]. Filtering should be used with care as it may also remove valuable information from the signals.

**Segmentation.** Discrete data points produced by the sensors are partitioned into time windows labeled from the most frequent activity in the window. The number of data points in a time window with a given sampling rate, i.e., window size, heavily impacts the performance of the model [4,15]. Finding the optimal window size depends on the specific requirements of the HAR system, e.g., the number of activities for which the system is devised or the maximum allowed latency for activity recognition [4]. However, in any case, the window size should be properly selected in such a way that each window contains enough samples (at least one cycle of an activity) to be differentiable from similar movements [3]. The current method to select the window size is empirical [15]. People apply ARP with different window sizes, which have been mostly selected from the values used in previous work, and they choose the one, which maximizes the performance of the recognition system. This process can be very time consuming due to the fact that there is no prior knowledge about the optimal window size and the whole space should be searched in an uninformed way.

**Feature extraction.** Each time window is then transformed into a vector of features, such as auto-correlation features [9], or statistical moments. These features are then used to help discriminate among various activities.

**Classification.** Finally, a classifier is trained on the vector of features and corresponding labels and assigns future observations to one of the learned activities. According to [5], DT, NB, SVM, KNN, Hidden Markov Models and ensemble classifiers such as Random Forest are the most important classifiers in HAR. 

The window size in the segmentation step and the feature selection in the feature extraction step are hyperparameters of the ARP, usually selected by trial and error as in [4].

### 2.2. Sliding Windows Technique

**Figure 3:**
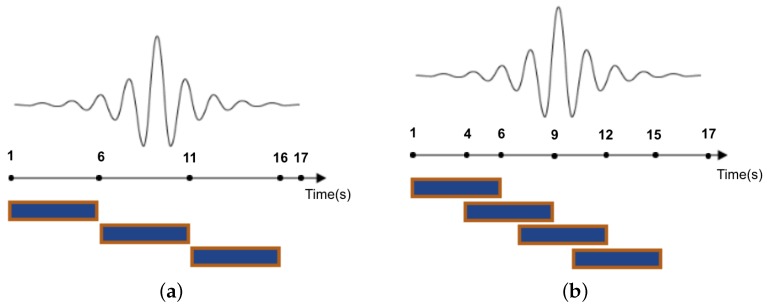
5 s sliding windows. (**a**) Non-overlapping; (**b**) Overlapping-2 s sharing.

In the segmentation step of ARP, the data points are partitioned into segments of data to capture the dynamics of activities. This process assumes that each window is an approximation of the signal for which the classifier will have to make a decision. There are several ways to segment the sensor signals in the HAR field which can be categorized into three groups, namely activity-defined windows, event-defined windows and sliding windows [4]. The sliding window approach is the most widely used method in the segmentation step of ARP. In this approach, the sensor signals are split into windows of fixed size. If there is overlap between adjacent windows, this technique is known as overlapping sliding window, and if not, it is called the non-overlapping windows technique. Figure 3 illustrates the non-overlapping and overlapping windowing techniques.

It is generally assumed that due to the higher number of data points, overlapping sliding windows increase the performance of HAR classifiers compared to non-overlapping ones [20], and they are not prone to missing important events [21], particularly within activity transition periods. While these assumptions are generally true, we will show later with our detailed experiments that non-overlapping windows deliver comparable recognition accuracy, while majorly reducing the required training computations and memory usage.

### 2.3. Formal Model

Mathematically, a sliding window process can be defined as follows. Assume a stream of data values $x_{i}\in R$ at times $t_{i}\left(i\in N\right)$. For simplicity and without loss of generality, we assume that $t_{0}=0$ and the sampling period remains constant at ΔT, i.e.,

$$\forall i\in N,t_{i+1}-t_{i}=\Delta T.$$

A fixed length sliding window splits the data stream into individual segments, where each segment consists of *n* (n∈N,n>1) samples. Consequently, the window size *T* in seconds is computed as follows:

T=(n−1)ΔT,

where ΔT is the sampling period. We also denote p∈{0,1,2,⋯,n−1} as the number of samples that are within the overlapping period between two consecutive windows, where *p* = 0 refers to the scenario with non-overlapping windows. Next, the overlapping period between two consecutive windows in seconds, i.e., OP, can be computed as follows:

OP=pΔT.

Many research articles define the overlapping period as a percentage of the overall window length, e.g., 80% overlapping windows. The overlapping period in percentage can also be found as follows:

$$OP\left(\%\right)=\frac{p}{n}$$

Finally, we can express each window/segment $S_{k}\left(k\in N\right)$ as a set of data values $x_{i}$ as follows:

$$S_{K}=\left\{x_{k(n-p)},x_{k(n-p)+1},\cdots ,x_{k(n-p)+n-1}\right\},\left(k\in N\right)$$

### 2.4. System Evaluation

**Figure 4:**
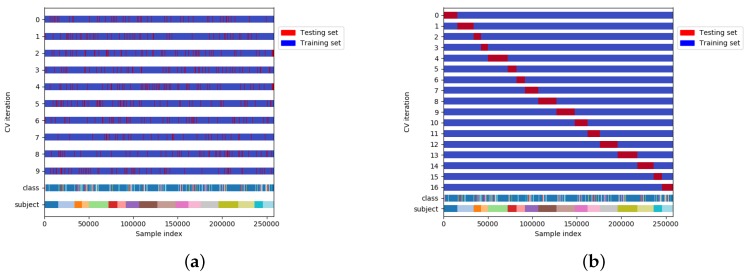
Different types of CV in HAR. (**a**) Subject-dependent CV; (**b**) Subject independent CV.

One of the most important steps in designing each system is evaluation. The evaluation in HAR has been mostly carried out through *k*-fold CV. In *k*-fold CV (Figure 4a), the overall data is randomly partitioned in *k* equal subsets. The model is then trained on k−1 subsets, and the remaining one is used for testing [22]. In this process, the test set can be any part of the dataset meaning that training and test sets may contain the data of the same subject and due to that, this method is referred to as subject-dependent CV in the literature [23]. The main assumption of this process is that *samples are Independent and Identically Distributed (i.i.d.)* [17], which means that all the data points are sampled independently from the same distribution. However, samples drawn from a given subject are likely to *not* be independent, for two reasons. First, there is a strong inter subject variability in the way activities are conducted [15]. This means that the similarity of samples drawn from the same subject is likely to be higher than that of samples drawn from different subjects. Several factors might explain such variability, including sex, gender, age or experience. Second, there is a temporal dependence between activities performed by the same subject: the similarity between samples drawn in a short time interval, for instance in the same training session in case of training activities, will most likely be higher than that of samples drawn further apart in time. This is due to factors such as fatigue and training. Thus, *k*-fold CV may overestimate the performance of recognizer systems in HAR. Such overestimation is even larger when *k*-fold CV is used with overlapping sliding windows since the overlap between adjacent windows is another source of dependency between data points. A more formal discussion about the problems of *k*-fold CV in HAR can be found in [24].

To address these issues, the training and testing sets should be split by subject. In this method, which is known as subject-independent CV [3,23], in each iteration the model is trained on all the subjects except one, which is used for testing. In this way, the intra subject dependencies present in subject-dependent CV are hence removed. It should be noted that, in this case, as is shown in Figure 4b, the number of folds is lower or equal to the number of subjects in the dataset.

## 3. Datasets and Experimental Setup

### 3.1. Datasets

In this study, we use two public datasets of human activity, to evaluate the impact of overlapping windows in a wide range of subjects, activities, and conditions.

**Dataset 1.** As the first dataset, we use the dataset described in [16], one of the most complete public datasets for HAR in terms of the number of activities and subjects. The dataset consists of data collected from 17 subjects of diverse profiles while wearing 9 Xsens [25] inertial measurement units (IMU) on different parts of their body. Subjects performed 33 fitness activities (Table 1) ranging from warm up to fitness exercises in an out-of-lab environment. Each sensor provides tri-directional acceleration, gyroscope, and magnetic field measurements, as well as, orientation estimates in quaternion format (4D). Similar to the prior work in [4], acceleration data was used in our study. The dataset also provides data for three sensor displacement scenarios namely “default”, “self-placement” and “mutual-displacement” to compare the sensor anomalies, but as in [4], only the data from default scenario is used in our study.

**Dataset 2.** The second dataset used is also one of the most complete and big public datasets for HAR in terms of the number of activities and subjects [9]. The dataset contains data for 74 activities (Table 2) from 94 subjects (28 female), ages 18–58 (μ=34.2). Data was collected in a large lab space from an armband worn on the right forearm, containing a SparkFun “Razor IMU” inertial sensor [26]. This device includes a 3-axis accelerometer and a 3-axis gyroscope and it can transmit sensor values to a PC at 50 Hz sampling rate. As can be seen in Table 2, there are several activities in the dataset such as “Arm band adjustment” and “Device on Table” which we consider as noise in this study. Besides, since during the data collection, the subjects wore a single joint sensor on their right forearm, the activities of the opposite hand (left hand) can not be captured properly with the sensors. Thus, we have done a relabeling process to clarify the dataset. In this process, (1) we label all the irrelevant and opposite hand activities as a “Noise” class (2) all the activities that refer to multiple labels are grouped together as one exercise. Table 2 shows all the activities and their labels after the relabeling process.

### 3.2. Experimental Setup

Similar to the prior work in [4], we did not apply any pre-processing to the dataset. We used both overlapping and non-overlapping windows. Overlapping windows were sliding at 200 ms, with window sizes ranging from 0.25 s to 7 s in steps of 0.25 s. For instance, a 5 s window shared 4.8 s of data with the previous one. Given the constant value of the sliding duration (200 ms), using a set of different window sizes is equivalent to exploring the impact of various overlapping sizes on the performance of our HAR systems. For non-overlapping windows, we used the same settings as in [4]: disjoint windows with sizes ranging from 0.25 s to 7 s in steps of 0.25 s. We used the same feature sets as in [4], namely FS1 (mean only), FS2 (mean and standard deviation) and FS3 (mean, standard deviation, maximum, minimum and mean crossing rate). Finally, for the classification part, we used the following classifiers: DT, KNN (K = 3), NB and Nearest Centroid Classifier (NCC). We used these classifiers as implemented in scikit-learn 0.20 [27] with default hyperparameters. We have also investigated non-default hyperparameters in another experiment. Furthermore, to provide a comparison with the framework in [28], we have utilized time-domain histogram features and the AdamOptimizer algorithm in Tensorflow 1.12.0 to train a neural network classifier with Sigmoid and Softmax trigger functions in the hidden and output layers, respectively. Such experiments will be explained in details in Section 4.

To evaluate model performance, we used both subject-dependent CV and subject-independent CV. We use the F1 score as a performance measure due to its robustness in class imbalance. F1 score which reaches its best at 1 and worse at 0, is computed as follows:

$$F1=\frac{2\times (precision\times recall)}{\left(precision+recall\right)}$$

All the source code for the conducted experiments are available in our GitHub repository [14]. The repository contains the scripts to segment the datasets for different window sizes, feature sets and sliding window techniques. There is also a script for training and testing all mentioned classifiers on windowed datasets. Finally, it also contains code to reproduce all presented figures in this paper.

## 4. Results

In this section, the impact of the overlapping and non-overlapping sliding windows in HAR systems with Subject-dependent CV and Subject-independent CV for both datasets is evaluated. The experiments are categorized into global evaluation and activity specific analysis.

For each experiment, we report distributions of average F1 score values obtained across all validation folds. Each distribution contains 28 measures obtained for the different window sizes mentioned in Section 3.2. We selected this representation over summary statistics or confusion matrix as it provides a comprehensive and synthetic overview of classification performance over a range of window sizes, classifiers, and feature sets. In all the figures of this section, “O” and “NO” stand for overlapping and non-overlapping windowing, respectively. Non-overlapping windowing is represented in green, and overlapping windowing is in red.

### 4.1. Global Evaluation

In these set of experiments, we analyze the general impact of overlapping and non-overlapping windowing in HAR systems trough the average performance of models for different activities and window sizes.

#### 4.1.1. Experiment 1: Subject-Dependent CV

In this experiment, we apply non-overlapping and overlapping windowing with Subject-dependent CV and use it as a baseline for further evaluations.

We applied the ARP system as explained in Section 3.2, on the datasets described in Section 3.1. For each window size, we partitioned the dataset in non-overlapping and overlapping windows separately and extracted feature sets FS1, FS2, and FS3 in each window. We trained the classifiers on the resulting feature vectors and measured their average F1 score over 10-fold CV.

**Figure 5:**
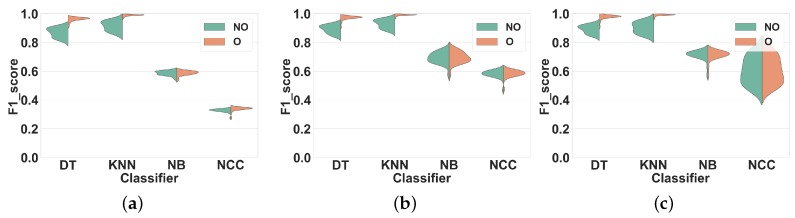
Experiment 1–Subject-dependent CV–Dataset 1. (**a**) FS1; (**b**) FS2; (**c**) FS3.

**Dataset 1.**Figure 5 shows the distribution of the F1 scores of the classifiers for different window sizes in overlapping and non-overlapping windows. The classifiers can be categorized into two groups: (1) KNN and DT, that have very different performance distributions for overlapping and non-overlapping windowing, and (2) NB and NCC, that show almost similar distributions for both techniques. Our findings show that, in general, using overlapping windowing improves the performance of all classifiers in all feature sets. Regarding the first group (KNN and DT), quantitatively, using the overlapping windowing technique improves the F1 score of the KNN and DT by about 10%, 8% and 8% on average in FS1, FS2, and FS3 respectively. However, the improvement for the second group is about 1%, on average, for all features sets, which is insignificant.

**Figure 6:**
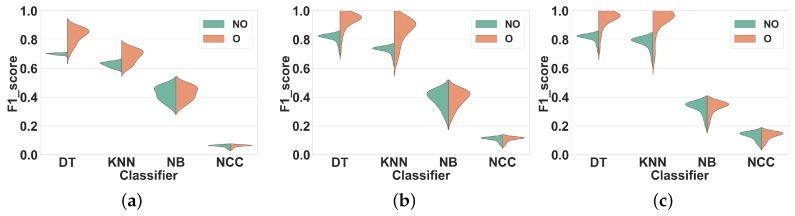
Experiment 1–Subject-dependent CV–Dataset 2. (**a**) FS1; (**b**) FS2; (**c**) FS3.

**Dataset 2.** The distribution of F1 scores for different window sizes and classifiers for overlapping and non-overlapping windowing is shown in Figure 6. Generally, the trends for Dataset 2 and Dataset 1 are similar. Overlapping windowing increases the F1 score and we observe the same two performance groups as before. The F1 score distributions of DT and KNN for overlapping and non-overlapping windowing are very different. Quantitatively, using overlapping sliding windows increases the performance of KNN and DT by about 9%, 12% and 13% on average in FS1, FS2, and FS3 respectively. Regarding the NB and NCC, however, the increase is minor to negligible for all feature sets. 

These results show that using the overlapping windowing technique rather than the non-overlapping one in subject-dependent CV improves the performance of classifiers. This agreement between our results and the general argument of the effectiveness of overlapping windowing in HAR systems [3,20] reinforces our confidence in the correctness of our analysis method and its applicability to our next experiments.

#### 4.1.2. Experiment 2: Subject-Independent CV

As explained in Section 2.4, subject-independent CV should be used to evaluate the performance of HAR systems. Thus, in this experiment, we compare the overlapping and non-overlapping windowing techniques when using subject-independent CV. The only difference between this experiment and Experiment 1 is the use of subject-independent CV rather than subject-dependent CV.

**Figure 7:**
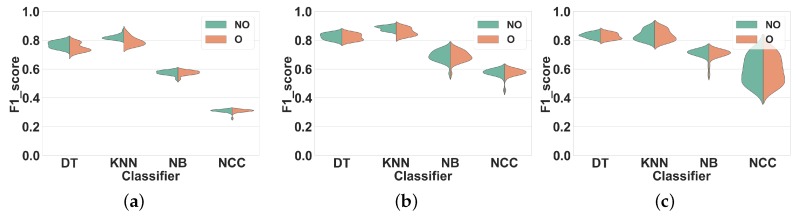
Experiment 2–Subject-independent CV–Dataset 1. (**a**) FS1; (**b**) FS2; (**c**) FS3.

**Dataset 1.**Figure 7 shows our results. Similar to Experiment 1 for this dataset, we observed the same two performance groups among classifiers. Regarding the first group (KNN and DT), however, overlapping windows do not lead to any improvement of the F1 score compared to non-overlapping windows. Overlapping windows even slightly decrease the F1 scores of DT and KNN in all feature sets, on average by 2% (FS1), 4% (FS2) and 1% (FS3). To further illustrate this result, Table 3 shows the F1 score of DT and KNN for several window sizes in overlapping and nonoverlapping windowing techniques for FS3: the performance of overlapping and non-overlapping windows is very similar. For NB and NCC the F1 scores obtained with both techniques are similar for all feature sets.

**Figure 8:**
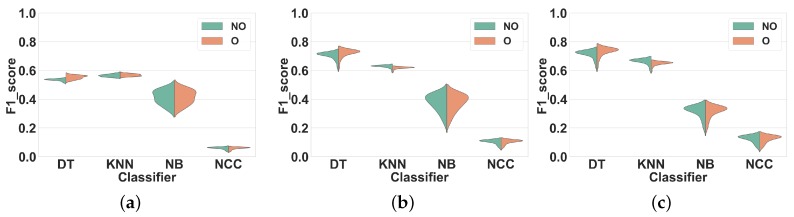
Experiment 2–Subject-independent CV–Dataset 2. (**a**) FS1; (**b**) FS2; (**c**) FS3.

**Dataset 2.**Figure 8 shows the results of Experiment 2 for Dataset 2. Once again, the F1 scores obtained for overlapping and nonoverlapping windows are very similar. This time, with DT and KNN, overlapping windowing improves the F1 scores slightly compared to non-overlapping windowing, on average by 2% (FS1), 1% (FS2) and 1% (FS3). The comparison between the performance of KNN and DT for several window sizes is shown in Table 3. For NB and NCC the F1 scores obtained with both techniques are similar for all feature sets.

In general, compared to Experiment 1 and for both datasets, the performance of all classifiers in all feature sets have decreased. This is most likely due to the fact that subject-independent CV removes the performance improvement overestimation associated with the use of overlapping windows under subject-dependent CV. This experiment confirms that the performance advantage of overlapping windows reported in the literature seems to have resulted from the use of subject-dependent CV and in case of using subject-independent CV, this method does not offer major benefits to the performance of HAR systems. Hence, we can reach to the same recognition performance by using non-overlapping windows. Considering the resource-intensity of overlapping windowing compared to the non-overlapping one, this is an important finding, since through that we can save a lot of resources (energy, time, memory, etc.), which is a desirable feature in HAR [5].

#### 4.1.3. Experiment 3: Subject-Independent CV and New Hyperparameters

One may claim that the results of Experiment 2 are due to the specific set of hyperparameters used in the classifiers. Thus, to investigate that, we reproduced Experiment 2 with a new set of hyperparameters for the KNN and DT classifiers.

We selected new hyperparameter values such that (1) overfitting or underfitting does not occur, and (2) the new values are as different as possible from those in the previous experiments. Table 4 compares the selected values for hyperparameters of DT and KNN in Experiments 2 and 3.

**Figure 9:**
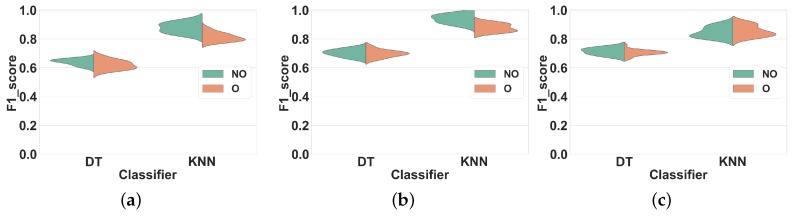
Experiment 3–Subject-independent CV–Dataset 1. (**a**) FS1; (**b**) FS2; (**c**) FS3.

**Dataset 1.** Results are shown in Figure 9. As in the previous experiments, the F1 scores obtained with overlapping and with non-overlapping windows are comparable. For DT, using overlapping windows decreases the performance in all feature sets, by about 1%. As for KNN, using overlapping windowing reduces the F1 score by about 4% in FS1 and FS2, and increases it by 1% in FS3.

**Figure 10:**
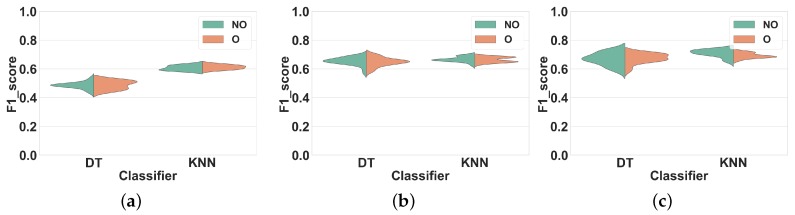
Experiment 3–Subject-independent CV–Dataset 2. (**a**) FS1; (**b**) FS2; (**c**) FS3.

**Dataset 2.**Figure 10 shows our results for this dataset. The trend is similar to the previous experiments, i.e., F1 scores obtained with overlapping and with non-overlapping windowing are very similar. Qualitatively, such differences for both classifiers remain lower than 1% in all feature sets. 

In conclusion, overlapping windowing does not provide any performance improvement compared to non-overlapping windowing with our new set of hyperparameters, which confirms the findings of Experiment 2.

### 4.2. Activity-Specific Evaluation

The global evaluation presented previously is useful to have a general view of the effect of windowing techniques in HAR systems. However, it is also interesting to particularize this study to each specific activity. Thus, in this section, we analyze the impact of overlapping and non-overlapping windowing for each activity.

#### 4.2.1. Experiment 4: Subject-Independent CV Per Activity

As shown by Experiment 2, overlapping and non-overlapping windowing techniques only lead to minor performance differences when evaluated with subject-independent CV. In this experiment, we investigate how this result particularizes in specific activities. The presented data is the same as in Experiment 2, but the classification performance is now detailed for each activity. For brevity, we only focus on feature set FS3. In all figures, activities are shown with the labels reported in Table 1 (Dataset 1) and Table 2 (Dataset 2).

**Figure 11:**
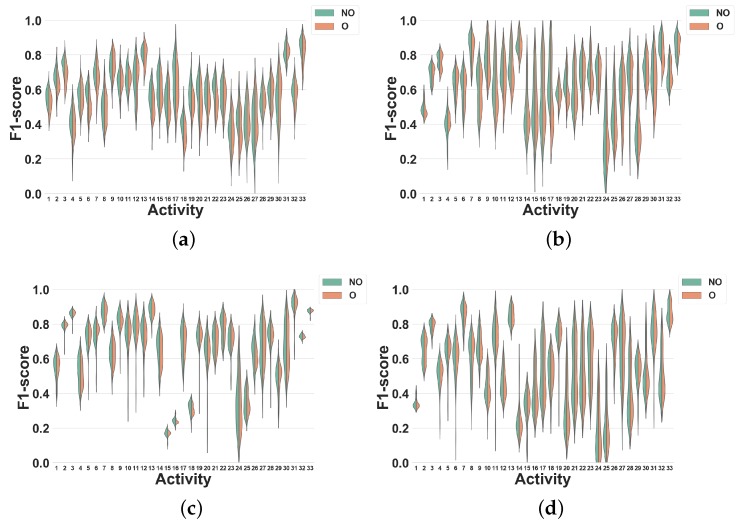
Experiment 4–Subject-independent CV–Dataset 1. (**a**) DT; (**b**) KNN; (**c**) NB; (**d**) NCC.

**Dataset 1.** In Figure 11, the activity specific F1 scores distributions achieved for all classifiers, window sizes and windowing approaches are presented. As expected from the results shown in Figure 7c, the differences between overlapping and non-overlapping windowing for all activities are low. In general, the performances of the majority of the activities drop (by 5% on average) when overlapping windowing is used instead of non-overlapping windowing. However, using DT (Figure 11a), some activities such as Heels (27), Rotation on knees (30), Trunk Twist (10), Knees (altering) (26), Knees Bending (28), Rowing (31) and Jump rope (8) show a small performance improvement by overlapping windowing. As for KNN (Figure 11b), performance reductions resulting from the use of overlapping windowing are higher than for DT. As an example, using overlapping drops the performance of activity Repetitive forward stretching (17) by 11%. This may be due to the nature of KNN [29], for which a small change in the dataset may have a large impact on the performance. Similar to DT, the performance for some activities also improves when overlapping windows are used, but by less than 2%. As can be seen in Figure 11c,d, the performance of all activities for NB and NCC are almost the same.

**Figure 12:**
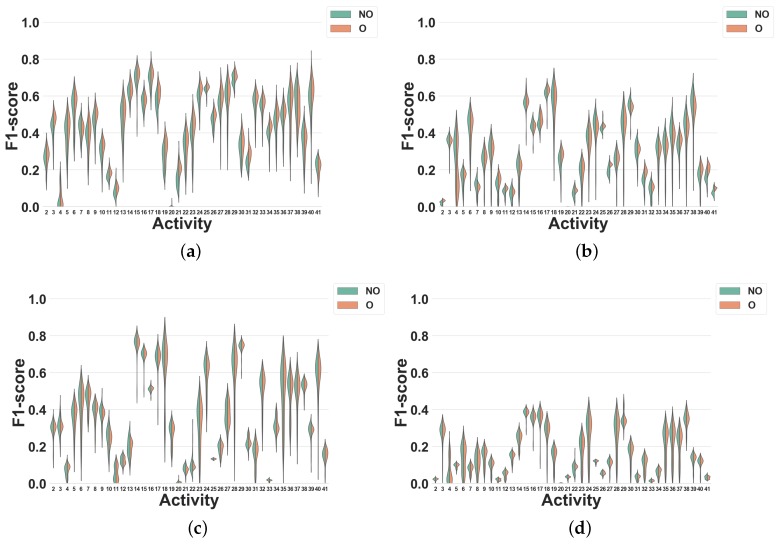
Experiment 4–Subject-independent CV–Dataset 2. (**a**) DT; (**b**) KNN; (**c**) NB; (**d**) NCC.

**Dataset 2.** Our results for this experiment are shown in Figure 12. 

This experiment shows that using overlapping windowing with subject-independent can impact the recognition performance of HAR systems for diverse activities differently. In spite of being mostly minor, for the activities in this study, overlapping windowing reduces the recognition accuracy of the most activities and only a few of them show improvement. Moreover, the impact of overlapping windowing may be subject to the dataset, i.e., using overlapping windowing may impact the performance of the system in recognizing a single activity in a different way. Running is a good example here. Using overlapping windowing reduces the F1 score of the system for this activity in Dataset 1, but it improves that in Dataset 2.

In summary, the recognition accuracy for most of the activities investigated in this study is quite comparable between the two scenarios with overlapping and non-overlapping windows.

#### 4.2.2. Experiment 5: More Discriminative Features and Neural-Network Classifier

In this experiment, we evaluate the effectiveness of non-overlapping windows using more discriminative time-domain features and a custom fine-tuned classifier. Namely, we target the HAR framework presented in [28]. The approach in [28] utilizes configurable time-domain histogram features and a single hidden layer neural network classifier. It has been shown that the framework can outperform KNN classifiers as well as many other time-domain features explored by previous work. The framework in [28] allows for segmentation with a configurable sliding window. It presents results on subject independent cross validation using Dataset 2 and a 5 s overlapping window sliding at 200 ms steps. Here, we conduct a similar experiment, but with a non-overlapping window size of 5 s. To provide a fair comparison, we use the same experimental setup compared to [28], which is presented next.

We use time-domain histogram features, i.e., 120 bins in total, where 20 bins are assigned uniformly to each individual sensor axis. The accelerometer full scale range has been set to ±2 g, while the gyroscope full scale range has been set to ±512 dps. The single-hidden-layer neural network classifier consists of 120 input neurons, 60 hidden neurons and 7 output neurons targeting 6 activities and one noise class representing all other activities and the no activity periods. The Sigmoid (Softmax) trigger function has been used for the hidden (output) layer. Training the neural network has been done in Tensorflow 1.12.0 using a batch size of 32, and the AdamOptimizer algorithm. The number of epochs is chosen, such that the overall number of data points that are fed to the neural network for training becomes identical compared to the experiment in [28], i.e., similar training time.

The normalized confusion matrix for both overlapping and non-overlapping windows under a subject-independent CV process is shown in Table 5. The rows refer to the true activities, while the columns correspond to the predicted ones. Each entry in the confusion matrix has two values, where the top value refers to the scenario with overlapping windows, while the bottom value corresponds to the scenario with non-overlapping windows. The results indicate that the use of overlapping windows provides minor improvements on recognition accuracy compared to the non-overlapping windows under subject independent cross validation, even when discriminative features and a well-trained neural network classifier are utilized.

## 5. Discussion

As can be seen by comparing the results of Experiments 1 and 2 for non-overlapping windowing, using Subject independent CV instead of Subject-dependent CV reduces the F1 score of KNN and DT by 10% and 11% on average for Datasets 1 and 2 respectively, which is substantial. It confirms that samples drawn from the same subject cannot be considered independent. In an ARP setup, Subject dependent CV overestimates the classification performance and should, therefore, be avoided. The detrimental effect of subject-dependent CV is even larger when overlapping time windows are used. In this case, as can be seen by comparing the results of Experiments 1 and 2 for overlapping windowing, Subject-independent CV reduces the F1 score of KNN and DT by 16% and 21% on average for Dataset 1 and 2 respectively. This further confirms that within subject dependencies between time windows account for a significant part of the performance measured through Subject-dependent CV. Furthermore, for overlapping windows, the performance difference between subject-dependent CV and subject independent CV increases with the window size. This is consistent with our previous comments, as the amount of overlap between overlapping windows, and therefore, their correlation also increases with the window size.

Comparing the results for overlapping and non-overlapping windowing in Figure 5 and Figure 6 shows that when using Subject-dependent CV, overlapping windowing can improve the recognition power of the classifiers, which coincides with the general idea that overlapping windowing improves the performance of HAR systems [3,20]. However, our results confirm that such improvement comes from increasing the correlation among test and train folds due to the underlying problems of Subject-dependent CV in HAR systems. In contrast, when using Subject independent CV, the impact of using overlapping windows is minor to negligible, as can be seen in Figure 7 and Figure 8. This is in contradiction with the common argument that overlapping windows improve classification performance by bringing more data to the classifier. However, it also confirms our hypothesis that the performance increase coming from overlapping windows is, in fact, coming from the extra correlation between time windows, when Subject-dependent CV is used.

Experiment 3 showed that this conclusion also holds when using a different set of hyperparameters, which improves the generalizability of our result.

The results of Experiment 4 show that the impact of overlapping windowing with subject independent CV can be different per activity. In other words, overlapping windowing for some activities such as Trunk Twist and Lateral Raise improves the recognition performances and for others like Repetitive forward stretching and Heels not. However, such changes remain negligible for most activities and using this technique in HAR seems to be non-beneficial.

Experiment 5 explored the use of more discriminative features with a neural-network model, and the results similarly suggest that the use of overlapping windows does not provide major performance improvements.

Finally, Table 6 shows the data size and required time for segmentation and training in overlapping and non-overlapping windowing techniques with subject independent CV for two datasets. Segmenting using overlapping windows is almost twice longer than with non-overlapping windows, which is significant. Similarly, the training time on the data windowed by overlapping windows is four times longer compared to the non-overlapping one. As for storage, the size of segmented data by overlapping sliding windows is almost nine times higher compared to the data produced by non-overlapping windows for both datasets. In spite of such increase in size and computation, this technique does not improve the performance of the classifiers when used with Subject independent CV.

## 6. Conclusions

We conclude that the suggested use of overlapping sliding windows in HAR systems is associated with underlying limitations of subject-dependent CV. When subject-independent CV is used, overlapping sliding windows do not improve the performance of HAR systems but still require substantially more resources than non-overlapping windows.

Our results show that the performance of all classifiers drops when subject-independent CV is used rather than subject-dependent CV. One possible way to address this problem would be to use features that are more common among subjects with different characteristics. Thus, in our future work, we will design such features and investigate their impact on the performance of HAR systems. This would enable building more generalized systems with a limited number of subjects.

## Figures and Tables

**Table 1:** Activity set in dataset 1.

Activity	Label	Activity	Label
No activity	0	Upper trunk and lower body opposite twist (20x)	18
Walking (1 min)	1	Arms lateral elevation (20x)	19
Jogging (1 min)	2	Arms frontal elevation (20x)	20
Running (1 min)	3	Frontal hand claps (20x)	21
Jump up (20x)	4	Arms frontal crossing (20x)	22
Jump front & back (20x)	5	Shoulders high amplitude rotation (20x)	23
Jump sideways (20x)	6	Shoulders low amplitude rotation (20x)	24
Jump leg/arms open/closed (20x)	7	Arms inner rotation (20x)	25
Jump rope (20x)	8	Knees (alternatively) to the breast (20x)	26
Trunk twist (arms outstretched) (20x)	9	Heels (alternatively) to the backside (20x)	27
Trunk twist (elbows bended) (20x)	10	Knees bending (crouching) (20x)	28
Waist bends forward (20x)	11	Knees (alternatively) bend forward (20x)	29
Waist rotation (20x)	12	Rotation on the knees (20x)	30
Waist bends (reach foot with opposite hand) (20x)	13	Rowing (1 min)	31
Reach heels backwards (20x)	14	Elliptic bike (1 min)	32
Lateral bend (10x to the left + 10x to the right)	15	Cycling (1 min)	33
Lateral bend arm up (10x to the left + 10x to the right)	16	-	-
Repetitive forward stretching (20x)	17	-	-

**Table 2:** Activity set in dataset 2.

Activity	Label	Activity	Label	Activity	Label
Arm band adjustment	1 (Noise)	Lawnmower (both)	20	Squat (arms in front)	33
Arm straight up	1 (Noise)	Lawnmower (left)	1 (Noise)	Squat (hands behind head)	33
Band Pull-Down row	2	Lawnmower (right)	21	Squat (kettlebell)	33
Bicep Curl	3	Lunge (both legs)	22	Squat Jump	33
Bicep Curl (band)	3	Ball Slam	23	Squat Rack Shoulder Press	33
Box Jump	4	No Exercise	1 (Noise)	Static Stretching	1 (Noise)
Burpee	5	Note	1 (Noise)	Stretching	1 (Noise)
Butterfly sit-up	6	Triceps Extension (standing)	24	Tap IMU	1 (Noise)
Chest Press	7	Triceps Extension (both)	24	Tap left IMU	1 (Noise)
Crunch	8	Plank	25	Tap right IMU	1 (Noise)
Device on Table	1 (Noise)	Power Boat pose	26	Triceps Kickback (bench–both)	34
Dip	9	Pushups (foot variation)	27	Triceps Kickback (bench–left)	1 (Noise)
Dumbbell Deadlift Row	10	Pushups	27	Triceps Kickback (bench–right)	34
Dumbbell Row (both)	11	Stretching	1 (Noise)	Triceps Extension (lying–both)	35
Dumbbell Row (left)	1 (Noise)	Rest	1 (Noise)	Triceps Extension (lying–left)	1 (Noise)
Dumbbell Row (right)	12	Rowing Machine	28	Triceps Extension (lying–right)	35
Dumbbell Squat (hands at side)	13	Running	29	Two-arm Dumbbell Curl (both)	36
Dynamic Stretch	1 (Noise)	Russian Twist	30	Non-listed	1 (Noise)
Elliptical Machine	14	Seated Back Fly	31	V-up	37
Punches	15	Shoulder Press	32	Walk	38
Invalid	1 (Noise)	Side Plank (left)	25	Walking lunge	39
Jump Rope	16	Side Plank (right)	25	Wall Ball	40
Jumping Jacks	17	Sit-up (hand behind head)	6	Wall Squat	41
Kettlebell Swing	18	Sit-up	6	Dumbbell Curl (alternating)	36
Lateral Raise	19	Squat	33		

**Table 3:** F1 scores of DT and KNN for several window sizes in overlapping (O) and nonoverlapping (NO) windowing–Subject-independent CV–FS3.

Dataset	Window Size (sec)	Classifier	F1-Score-O (%)	F1-Score-NO (%)
1	1	DT	86.42	86.0
		KNN	89.04	88.78
	4	DT	81.97	83.38
		KNN	82.05	82.18
	6	DT	80.1	80.83
		KNN	79.08	79.30
	7	DT	80.61	80.62
		KNN	77.74	78.68
2	1	DT	69.26	67.38
		KNN	63.61	63.94
	4	DT	74.37	73.06
		KNN	65.52	67.01
	6	DT	75.28	73.12
		KNN	66.0	67.8
	7	DT	75.46	73.06
		KNN	66.0	67.93

**Table 4:** The hyperparameters values for KNN and DT in Experiments 2 and 3. When max_depth is set to None, the decision tree is expanded until all leaves are pure [27].

Classifier	Hyperparameter	Experiment 2	Experiment 3
KNN	K (n_neighbors)	3	6
DT	Criterion	‘gini’	‘entropy’
	max_depth	None	20
	max_features	1	0.7

**Table 5:** Normalized confusion matrix for Experiment 5, i.e., six activities and one noise class representing all other activities and no activity periods from Dataset 2 under subject independent cross validation. Rows are true activities, and columns are predicted ones. Each entry has two values, where the top (bottom) value refers to the scenario with overlapping (non-overlapping) windows.

Activities	Noise (Others)	Curl	Triceps	Run	Elliptical	JumpJacks	Kettlebell
Noise (Others)	**0.99407** **0.9928**	0.00094 0.00103	0.00091 0.00099	0.00209 0.00286	0.00127 0.00129	0.00026 0.00028	0.00045 0.00074
Curl	0.1426 0.1369	**0.85082** **0.8492**	0.00659 0.0139	0 0	0 0	0 0	0 0
Triceps	0.17235 0.19643	0.00451 0.00487	**0.82315** **0.7987**	0 0	0 0	0 0	0 0
Run	0.20903 0.20127	0 0	0 0	**0.78668** **0.79237**	0.00429 0.00565	0 0	0 0.00071
Elliptical	0.16742 0.16265	0 0	0 0	0.00916 0.03113	**0.82342** **0.80622**	0 0	0 0
JumpJacks	0.20147 0.23729	0 0	0 0	0 0	0 0	**0.79853** **0.76271**	0 0
Kettlebell	0.18938 0.21484	0.00287 0	0 0	0 0	0 0	0 0	**0.80775** **0.78516**

**Table 6:** Overlapping windowing vs. nonoverlapping windowing required resources-Subject independent CV.

Dataset	Raw Size (GB)	Nonoverlapping Windowing			Overlapping Windowing		
		Segmentation		Training Time (Day)	Segmentation		Training Time (Day)
-	-	Time (Hour)	Size (GB)		Time (Hour)	Size (GB)	
1	2.4	6.0	2.3	1.0	11.0	21	4.0
2	3.4	12.0	5.8	2.0	20.0	51	8.0
